# Nature and Potential Impact of Alcohol Health Warning Labels: A Scoping Review

**DOI:** 10.3390/nu13093065

**Published:** 2021-08-31

**Authors:** Daša Kokole, Peter Anderson, Eva Jané-Llopis

**Affiliations:** 1 Department of Health Promotion, CAPHRI Care and Public Health Research Institute, Maastricht University, POB 616, 6200 MD Maastricht, The Netherlands; peteranderson.mail@gmail.com (P.A.); eva.jane@esade.edu (E.J.-L.); 2 Population Health Sciences Institute, Newcastle University, Baddiley-Clark Building, Richardson Road, Newcastle upon Tyne NE2 4AX, UK; 3 ESADE Business School, University Ramon Llull, Avenida de Pedralbes, 60-62, 08034 Barcelona, Spain; 4 Institute for Mental Health Policy Research, CAMH, 33 Russell Street, Toronto, ON M5S 2S1, Canada

**Keywords:** alcohol, labelling, health warning labels, effectiveness, implementation

## Abstract

Alcohol is toxic to human health. In addition to providing nutritional information, labels on alcohol products can be used to communicate warnings on alcohol-related harms to consumers. This scoping review examined novel or enhanced health warning labels to assess the current state of the research and the key studied characteristics of labels, along with their impact on the studied outcomes. Four databases (Web of Science, MEDLINE, PsycInfo, CINAHL) were searched between January 2010 and April 2021, and 27 papers were included in the review. The results found that most studies were undertaken in English-speaking populations, with the majority conducted online or in the laboratory setting as opposed to the real world. Seventy percent of the papers included at least one cancer-related message, in most instances referring either to cancer in general or to bowel cancer. Evidence from the only real-world long-term labelling intervention demonstrated that alcohol health warning labels designed to be visible and contain novel and specific information have the potential to be part of an effective labelling strategy. Alcohol health warning labels should be seen as tools to raise awareness on alcohol-related risks, being part of wider alcohol policy approaches.

## 1. Introduction

Globally, 5.3% of all deaths and 5.0% of all disability adjusted life years are caused by ethanol [1], a toxic substance [2]. Alcohol consumption is causally related to more than 40 ICD-10 three-digit categories [3], including non-communicable diseases such as liver disease [4], cancers of the colon, liver and breast [5,6,7], alcohol use disorders [8], non-ischaemic cardiovascular diseases [9]; communicable diseases such as tuberculosis [10]; and intentional and unintentional injuries [3]. Alcohol can also impair mental health (particularly depression [11,12]) and cause harm to others, such as in foetal alcohol spectrum disorders [13,14].

Despite alcohol’s detrimental effect on a variety of health outcomes, public awareness about some health risks associated with alcohol consumption remains relatively low. A 2018 review of 32 studies found that while awareness of alcohol as a risk factor for cancer varies by country, in most studies, less than half of the respondents correctly identified the alcohol–cancer link [15]. This finding is confirmed in more recent studies [16,17,18], which additionally found that awareness of the link between alcohol and cancer differed by type of cancer (e.g., the link between alcohol and breast cancer tended to be the least well known).

One of the approaches to raise awareness of the health risks associated with alcohol consumption can be product labelling. A review from 2013 on enhanced labelling [19] identified five elements that could be useful to consumers: a list of ingredients, nutritional information, serving size and servings per container, a definition of moderate intake (low risk drinking guidelines) and a health warning label (HWL). While the majority of countries have mandated alcohol volume content on the labels [20], this is not the case with nutritional or health labelling. In many of the high consumption regions, such as EU countries, UK, US, Australia, New Zealand and Canada, alcoholic beverages are exempt from regulations to include nutritional information (for example, nutritional values and calorie content) on the product label [21,22,23,24], as opposed to non-alcoholic drinks and food products. Nutritional labelling policy thus leaves much room for improvement, with clear guidelines available on how information should be presented based on the requirements for food and non-alcoholic beverages should nutritional labels be mandated for alcoholic beverages. Whilst there is scarce alcohol-specific research on nutrition information [25,26], much is known from the nutrition field on enhanced presentation of nutritional information [27,28], which could be applied to alcoholic beverages.

There is little available evidence on the effectiveness of health warning labels that would inform consumers about the risks associated with alcohol consumption. Most older studies examining the effectiveness of implemented health warning messages report on the US health warning label, which focuses on risks surrounding drinking during pregnancy, and has not changed since its introduction in 1989 [29,30]. More recent studies on existing health warning labelling schemes, either mandatory [31,32] or voluntary [33,34,35], show that the existing labels (e.g., pregnancy logo or responsibility message) are suboptimal and are either not being noticed or not being understood. However, evidence from tobacco shows that health warning labels can be a very important part of the broader package of interventions [36,37], and there have been calls to apply tobacco-style health warning labels to alcohol products [38]. Given the evidence for the less effective existing alcohol HWL, this review focuses on examining evidence with regard to novel (not previously implemented in practice) or enhanced labels (improved versions of existing ones).

The importance of labelling is recognised by the World Health Organisation, whose global strategy to reduce the harmful use of alcohol [39] calls for providing consumer information about the harm related to alcohol and labelling alcoholic beverages to indicate such risks. Awareness of such information is, in turn, linked to increased public support for more stringent policies, such as taxation [40,41], and can therefore be an important part of effective alcohol policy. There has been increased interest among policymakers to provide health information to consumers through labels. Ireland passed the Public Health Alcohol Act in 2018, mandating cancer warnings on labels [42] and becoming only the second country in the world after South Korea so to do [20], but nothing else has been implemented as of 2021. There has also been increased research activity, and a number of labelling-related reviews have been published recently. Previous reviews focusing on alcohol HWL operated with a relative scarcity of studies [43], focused only on one aspect of the label (image vs. text) [44] or made a brief narrative overview of health labelling studies as part of broader alcohol labelling [45]. Whilst our work partially overlaps with other reviews in examining the impact of labels, our review adds a thorough and systematic examination of the scope of (quasi-)experimental research on new or enhanced health warning labels, including an overview of the labels used in the studies and the explanatory variables studied. As there is not a single definition of “alcohol HWL”, labels can differ in how they are developed, implemented and evaluated, and the current review aims to contextualise impacts in light of this information. As health warning labels, we consider labels that are containing information on the relationship between alcohol and health outcomes. We do not focus on drinking guidelines and standard drinks labelling, as this has been carried out elsewhere [46].

Thus, the aim of this scoping review is to examine the peer-reviewed literature studying novel or enhanced health warning labels and to answer two research questions: (1) what is the scope of the current alcohol HWL research (in terms of research aims, studied independent variables, the outcomes of interest, as well as label content and format), and (2) what is the impact of the labels and key label characteristics on the studied outcomes?

## 2. Methods

### 2.1. Design

To answer the research questions, we used scoping review methodology, guided by the methodological framework of Arksey and O’Malley [47]. Scoping reviews are used to map the main concepts in the research area and present a broad overview of the existing evidence, including identification of the gaps, regardless of the study quality [48]. The protocol was not published in advance, and the results are reported in accordance with PRISMA-ScR checklist [49].

### 2.2. Eligibility Criteria

To be included in the review, studies had to evaluate new or enhanced alcohol health warning labels in any population. By “new”, we considered labels developed for the purpose of the research and not previously implemented in practice, and by “enhanced”, we considered improved versions of existing labels already implemented in practice. Studies had to include experimental or quasi-experimental methodology with clearly delineated independent and dependent variables, in order to be able to synthesise results based on the manipulated variables and to identify the key characteristics of effective labels. Only peer-reviewed articles in the English language published in 2010 or after were included, as in the previous reviews, there were no older experimental studies of new messages. Studies that did not include health warning messages, that focused on qualitative data or were only cross-sectional evaluations were excluded.

### 2.3. Information Sources and Search Strategy

Four databases (Web of Science, MEDLINE, PsycInfo, CINAHL) were searched with the abovementioned restrictions on language and dates (English language, January 2010–April 2021). Additionally, we complemented the database searches with inspection of reference lists of relevant articles and reviews, and we undertook a Google Scholar search. The search strategy contained a combination of terms related to alcohol labelling: [alcohol AND (label OR label*) AND (message OR information OR warning) AND (experiment* OR eval* OR effect)].

### 2.4. Study Selection and Summary

Study selection was performed by one researcher according to the abovementioned inclusion and exclusion criteria, with ambiguous cases being resolved through consultation with the second researcher. After the removal of duplicates, the studies were first screened by title and abstract, followed by full text examination and final article selection. Next, the data were charted to answer the main research questions. Next to general information on the papers’ aim and methodology, information on label development and implementation was charted, as well as the independent studied variables and the results for each of the outcomes. Papers rather than studies were taken as the units of interest, as the focus of the review was to appraise the scope of the published literature and to identify results for all the studied outcomes.

## 3. Results

In total, we identified 27 papers that fit our inclusion criteria (see Appendix A for PRISMA diagram) [50,51,52,53,54,55,56,57,58,59,60,61,62,63,64,65,66,67,68,69,70,71,72,73,74,75,76]. One paper reported on two studies using different methodologies to study the same variables [50], and four papers reported on different aspects of one intervention [51,52,53,54]. The remaining papers were individual studies (with a complete or partial focus on alcohol HWL labelling), although some of them were part of the same research line of label development and testing (e.g., [55,56,57,58,59,60]) or studied the same labels (e.g., [61,62]).

Table 1 provides an overview of the papers with their aim and methodology. Two-thirds (18) of the included papers were published in 2018 or later, indicating an increasing research interest in the topic. All of the research was undertaken in Western countries: most papers reported on research conducted in the UK (nine), Canada (six) and Australia (five). The remaining papers reported on research conducted in the US (three), Germany (two) or were multi-country (twice Luxembourg/Germany, and once Italy/France). Only four papers described real-world interventions. The remainder of the studies were either conducted online (sixteen) or in a laboratory (eight, of which one was in a naturalistic shopping laboratory). Six studies had under 100 participants, eleven studies had above 1000 participants and one study relied on sales data. The majority (80%) of the studies with a lower number of participants were conducted in the laboratory and before 2018, indicating that in recent years, larger sample sizes were accessible in an easier manner through online panels. Two-thirds of the papers focused on the general adult (of legal drinking age in the country) drinking population, although the exact inclusion criteria differed and ranged from people drinking at least once in the past 12 months, to people consuming alcohol weekly. Eight of the papers focused on the younger population/students (some by convenience rather than by inclusion criteria) and one paper focused on secondary school students.

### 3.1. Research Scope

To answer the first research question, we looked at the aim of the papers and the variables studied (both independent variables and the outcomes), as well as at the content and format of the studied labels.

In terms of study aims and studied independent variables, five papers (19%) compared only new or enhanced labels with no or regular labels [51,52,53,54,63]. Focusing on label content, eight papers (30%) compared text-only labels with text and image labels [55,56,61,62,64,65,66,67], twelve studies (44%) compared text message characteristics (e.g., content, specificity, framing, source, use of causal language) [57,58,59,60,67,68,69,70,71,72,73,74] and three (11%) compared image characteristics [57,66,75]. Four (15%) papers compared label formats, such as size, position, colour or branding [50,64,70,76]. Additionally, nine papers (33%) studied other variables, such as alcohol content, alcohol/substance type, efficacy information, expectancy and self-affirmation [64,65,66,68,69,73,74,75,76].

The majority of papers investigated more than one outcome (Table 2). The most commonly studied outcomes were attitude (41% of the papers), intentions (30%), emotion-related outcomes (e.g., fear, worry) (26%) and acceptability/support for the labels (26%). Behaviour was studied in six papers (22%)—in three of them through self-report [51,54,67], but also through purchasing [53,56] or consuming alcohol [62].

The tested labels were predominantly developed by the researchers themselves, based either on other research (e.g., related to tobacco and alcohol labels, alcohol harm, effective messages) or on own development and piloting (e.g., through qualitative studies), or both. In the four papers related to a real-world intervention [51,52,53,54], the labels were additionally (next to own and other research) developed in consultation with local stakeholders. In two cases, the labels used in the research were developed by a non-governmental organisation [67,75], and in one case [50], existing voluntary labels were enhanced based on theory.

In terms of label content, eight studies studied a single message; the remaining were either comparing different labels (or groups of labels) or used several messages as a single intervention. Most commonly, the messages were referring either to cancer in general (e.g., “Warning: Alcohol causes cancer”) [51,52,53,54,57,58,59,60,63,67,71,75], or specifically to bowel cancer (e.g., “Alcohol causes bowel cancer”) [51,52,53,54,55,56,57,58,59,60,69,71], both in 41% of all papers. Overall, 70% of papers included at least one cancer-related message, with other mentioned cancers being also breast (33%) [51,52,53,54,55,56,57,58,60] and liver cancer (30%) [55,56,57,61,62,64,69,76]. Liver cirrhosis or liver disease message was used in nine (33%) papers (e.g., “Warning: Alcohol causes liver disease”) ([56,57,59,65,66,67,72,73,75]. Heart disease [55,57,59,72], mental illness [59,67,71,75], brain damage [70,72,74,75] and drinking and driving-related messages [67,70,74,75] were used in four papers each (15%), and pregnancy messages in three papers (11%) [50,67,75]. Four papers (15%; [59,67,68,72]), included other topics of messages such as diabetes, positive expectancies related to alcohol, warning against operating machinery or serving alcohol to minors. Fifteen papers (56%) included images in the labels—ten of them included only graphic images (e.g., images of diseased body parts) [55,56,57,61,62,64,65,68,69,76], two included both graphic and neutral images [66,75], one included only neutral images [67] and two included pictograms [50,70].

In terms of label format, the majority of the papers formatted the labels to be clearly visible. This meant putting labels on the front of the pack, with a predetermined size and clear contrast between text and background and separation from the rest of the label (e.g., black text on white background with black or red border, such as [55,56,57,61,63] or black text on a bright yellow background and red border, such as [51,52,53,54]). A complete description of the studied labels content and format is available in Supplementary Table S1.

### 3.2. Key Label Characteristics—Impact on the Outcomes

Results for all the studied outcomes from each of the papers are presented in Table 3. Below, the results are summarised based on the study aims identified through the two research questions: (1) studying overall label effectiveness by comparing image and text labels and (2) studying other label message characteristics and label formats.

#### 3.2.1. Label Effectiveness

A large online UK study [63] did not find that adding a text warning to alcohol drink label changed perceived personal risk, motivation to drink less or perception of damaging drinking among adult drinkers. On the other hand, the Canadian real-world study, which included putting three types of labels (one of them a cancer warning) on the majority of alcohol drinks in the participating liquor stores in the studied region, found that these enhanced labels led to increased recall, cognitive processing and self-reported impact of drinking [51,54], as well as decreased alcohol sales [53] in the intervention municipality compared to the control municipality. While the whole intervention included both cancer warnings as well as low-risk guidelines/standard drink labels, it is not possible to completely disentangle the impact of solely the cancer warning labels from the results, although some results pointed to effects being driven predominantly by the cancer label. The one paper that looked specifically at the cancer labels [52] found that cancer labels increased knowledge of alcohol as a carcinogen, and that knowledge increased with time. Additionally, the authors found that knowledge about alcohol causing cancer was associated with a greater likelihood of supporting health warning labels.

#### 3.2.2. Label Content—Image vs. Text

Among the eight papers that compared image and text labels, six had control group comparison [55,61,62,64,67] and in two [65,66], only text and image were compared. Six of the papers used graphic images (e.g., diseased body part); in one, the images were neutral, and one contained both graphic and neutral images.

In the studies where graphic images were used, comparison between text-only and image-and-text labels showed higher negative emotional arousal [55,56], fear [55,56,61,65] and reactance [65] for the image-and-text labels among adults compared to text only labels, as well as lower product appeal [64,65], label acceptability [55,56] and believability [65]. Moreover, in the study using neutral images [67], there were higher negative emotions for image-and-text labels compared to text-only were found among the secondary school students, but only for some messages. The same study also found that both types of labels are better than no label for increasing knowledge about alcohol-related risks when knowledge is low at baseline. While an online experiment showed a selection of alcoholic drinks in the given scenario to be lower after seeing image-and-text labels compared to text-only labels [55], this finding did not replicate in a naturalistic lab shopping setting [56] among adult regular drinkers, where none of the labels affected purchasing behaviour. In another study, both text-only and image-and-text labels decreased the speed of consumption compared to no label, with no significant difference between them [62].

The two studies actively looking at the differences between graphic and neutral images did not find any difference between the two in attention [66,75], although the latter found higher avoidance, reactance, perceived warning effectiveness and motivation to drink less in graphic compared to neutral images.

#### 3.2.3. Label Content—Message Characteristics

Several papers also compared different messages or their characteristics. In terms of message *content*, in Australia, one study among adult drinkers found increased intention to reduce drinking for cancer, diabetes and mental illness messages, but not for heart disease and liver damage-related messages [59]. Another study found that a bowel cancer message had the highest believability, convincingness and personal relevance [60]. A bowel cancer message also had the highest negative emotional arousal, lowest desire to consume the labelled product and lowest acceptability in a large-scale UK online study of adult drinkers [57], but in this study, all the labels included graphic photos, which might have influenced the outcome. Another large scale online experiment in the UK among adult drinkers [71] compared a cancer message to a mental health message and found the former led to a higher motivation to drink less and higher avoidance, but there was no difference in reactance, believability, self-efficacy and response efficacy. Another smaller study found that positive expectancy-related messages lowered drinking intentions compared to health-related messages among students from Germany and Luxembourg [72]. In a German sample of secondary school students, cancer and liver cirrhosis messages, but not a pregnancy label, increased knowledge about alcohol-related risks [67].

In terms of *framing*, the discrete choice experiments gave mixed results [70,74]; in the first study, no message at all was more preferred than any message in a sample of Italian and French young wine drinkers, and in the latter, the negative message was less preferred than the neutral message among Australian students. In an online study of British adult drinkers [71], a negative framing led to a higher motivation to drink less, with increased reactance and avoidance compared to a positive framing, but did not influence believability, self-efficacy and response efficacy. This study also looked at *specificity* and found that specific messages led to lower reactance, higher response efficacy and higher believability compared to general messages, but there was no difference in motivation to drink less, avoidance and self-efficacy.

One study looked at whether framing labels as questions or statements impacts attitude and intentions, and found no difference [68]. Another study compared narrative and non-narrative labels and found narrative labels led to higher worry about developing alcohol-related cancer and higher feelings of risk of developing alcohol-related cancer, but no difference in intentions to reduce alcohol use [69]. The study examining causal language [73] found that using the word “causes” had higher perceived message effectiveness, and “may contribute to” had the highest perceived ineffectiveness, but there was no difference in support for any of the labels. Finally, a study of Australian adult drinkers found that receiving the message from multiple sources is better than receiving a message from one single source [58].

#### 3.2.4. Label Format

Finally, four studies examined label format: branding/plain packaging [64,76], warning size [50,70,76] and warning position [70]. Two smaller-scale Canadian studies found that plain packaging (removing any branding) decreased product-based and consumer-based perception [64,76]. The latter study [76] also found that after a certain size of the warning (50% of the bottle), there is no further increased difference in outcomes. In a discrete choice experiment, young Italian and French wine drinkers preferred to have warning sizes as little as possible, and on the back of the label rather than on the front of the label [70]. Finally, one study [50] found that an increase in size and change in prominent colour was effective in self-reported measures of attention among the Australian drinkers, but not when measured directly with eye-tracking.

## 4. Discussion

This review aimed to examine the scope of the (quasi-)experimental research on novel and enhanced alcohol health warning labels and to identify the key characteristics of the effective warning labels. Twenty-seven papers were included, the majority of which were published in 2018 or later, indicating an uptake in interest surrounding the topic from the research community (recognised also in other broader reviews [45]). This is aligned with the increase in policy-related labelling discussions, such as in Codex Alimentarius, at the EU or national level (e.g., [77,78]). All of the research has been undertaken in Western countries, where alcohol labelling has either not been updated since its implementation decades ago (e.g., Canada, US), carried out on a voluntary basis (e.g., Australia, UK) or not mandated at all (e.g., many of the EU countries). Given high alcohol consumption in those regions [79], this may reflect the recognition of labelling of potentially effective means of reducing alcohol-related harm—although it is debatable if this focus adds to or replaces focus on “best buys” policies [80], such as taxation, pricing and advertising restrictions. In addition, the majority of the studies focused on general population, which reflects a shift from findings of previous review [43], where less than a third of studies were targeting the broader population, and the remaining were targeting younger populations and (pregnant) women.

In terms of content, most commonly, the studied messages were referring to cancer—70% of papers included at least one cancer-related message, out of which the most commonly mentioned cancers were bowel, breast and liver cancer. This was followed by liver cirrhosis or liver disease messages, used in a third of papers. Other topics of messages were heart disease, mental illness, brain damage, pregnancy, drinking and driving and other (e.g., positive expectancies surrounding alcohol). This brings us to the question on how to select the content of the message to be included on the labels. In the papers included in this review, researchers have commonly either developed messages based on “evidence on alcohol-related harm” or have taken messages used in other studies. We would argue that the message first has to reflect evidence-based risk (e.g., that causal relationship between alcohol and the outcome has been clearly demonstrated [3]), and then the message should be tailored to the context in which the labels will be displayed, based on the burden of disease. Another factor that has been found to be important for the selection of the message is also its novelty for the given audience [16,53]. For example, in the EU, 29.4% of all alcohol-attributable deaths and 19.4% of all attributable DALYs are due to cancers [81], while awareness of the risk between alcohol and cancer is low [15]. Based on these guidelines we can conclude that cancer warnings should be a priority when mandating health warning labels in this region.

Next, the focus of research implies the researcher’s theory of change—the mechanisms by which they consider labelling to work [82]—and this will influence the targeted outcomes and how effectiveness is measured. This is relevant because the policy debate will inevitably discuss the merit of the health warning labels based on their “effectiveness”—previously, one of the arguments in the World Trade Organisation’s discussions was that labels are considered unnecessary interference with international trade because there is no scientific evidence to support such warnings as an effective public health measure [83]. Based on the scope of the existing research, we propose that two possible theories of how labelling brings about change are currently being investigated. The first one is focused on persuasion and short-term individual behaviour change, and optimises using health warning labels that aim to elicit reactance, avoidance, fear and other negative emotional reactions as means of changing behaviour, drawing heavily on the experience from tobacco warning labels. For this line of research, there is no real-world evidence from the alcohol field, and mixed evidence from lab studies regarding (short-term) behaviour [56,62]. The (online and lab) studies that focused on studying the graphic images on labels found that they elicited higher negative emotional arousal, fear and reactance, and were not well accepted by the participants. These results are also corroborated by the metanalysis investigating the impact of HWL in both alcohol and food products, and found that while health warning labels reduce product selection compared to no labels, the difference between image-and-text HWLs and text-only HWLs was not statistically significant (although in the former, the effect was slightly larger) [44].

The second theory of change sees labelling as a tool to raise awareness of alcohol-related risks as a part of a comprehensive strategy to reduce alcohol-related harm (which should include other alcohol policies and interventions), and emphasises optimising labels to increase attention to and cognitive processing of the information on the labels, leading to increased knowledge (e.g., on alcohol health risks). This approach is more in line with system perspectives [84] and its focus is to use health warning labels to arrive at a better informed population that could in turn lead to more support for other alcohol policies. Individual short-term behaviour change might be a welcome by-product of this approach, but in the long term, labels can be seen as a tool to help with changing societal conversation and norms surrounding alcohol (as suggested also by O’Brien [85]). In terms of evidence, the best current evidence points to the second theory of change as having greater potential to be explored in future research and practice. The most comprehensive study to date and the only one measuring long-term effects in the real world [86] showed that simple, specific and well-visible messages led to an increase in knowledge (with associated support for stronger alcohol policies [87]) and change in the amount of alcohol consumed, partially mediated by the consumer attention to and processing of label messages [54]. The labels were also well accepted and supported by the population, as well as the drinkers [51].

It is important to note that this study [86] was implemented in context with existing relatively strict other policy measures—e.g., national monopoly on the alcohol stores (alcohol being sold separately in the government-owned liquor stores), and enhanced label replacing existing label (rather than no label). Nevertheless, a key approach to label development and implementation in this study is a good practice example and should be used in any future attempts to study alcohol HWLs in different contexts. This means developing labels in consultation with local stakeholders, including a range of different messages (not only health but also related to standard drinks and lower-risk guidelines) and including evidence-based messages for which there was low awareness at baseline and are relevant to the population. This study [83] also points to the unintended consequences that can be anticipated—originally, the study was planned to run for eight months with all three labels, but the alcohol industry intervened and threatened with a lawsuit if cancer warning labels were to continue being applied on the products [83]. Thus, cancer labels were only applied to the alcohol products for one instead of eight months. This shows that in any attempts to mandate evidence-based alcohol health warning labels, strong opposition from the industry can be expected, similar to what happened in the tobacco field [88].

Thus, while there should be some lessons from tobacco taken for alcohol health warning labels, we must be careful to still consider alcohol in its own context, and note that the evolution of alcohol legislation lags behind the tobacco field by a couple of decades. There is evidence for the effectiveness of pictorial labels for tobacco [36], but even here, the argument can be made that labels have been part of a broader approach that involved international collaboration and changed societal norms around tobacco [89], and their purpose was perhaps less to deter smokers from smoking than to indirectly prevent younger populations from taking up smoking, as tobacco warning labels replaced packaging as the last venue of marketing after other forms of advertising had been prohibited [38]. The value of adding images on alcohol labels might be to help with attracting attention and take away space from the branding of the product, but not enough studies with only neutral images or symbols have been conducted to make any firm conclusions.

The main implications for research and policy based on the results of this review are summarised in Table 4. We would like to note that the same theory of change useful for health warnings could also be useful to develop and test for optimal nutrition information on alcohol labels (not direct focus on changing behaviour, but focus on providing the information), although as previously mentioned, the issue with nutritional labels is to first align requirements for alcoholic beverages with existing labelling requirements for food and non-alcoholic beverages.

### 4.1. Limitations

Our review is subject to general limitations of scoping reviews: we did not appraise studies for quality, and the conclusions are still somewhat broad and qualitative. In terms of this specific review, we narrowed the scope of our investigation to health warning labels and overall outcomes and we did not focus on other labels, such as lower-risk drinking guidelines or standard drinks, even though some research suggests it might be worth taking them into account simultaneously. We also did not investigate differential impact of labels on different subgroups. Evidence from Canada points to some subgroup differences in cognitive processing of the labels, although concludes that vulnerable populations still attended to the labels [52]. Our search was also focused only on peer-reviewed English language papers; therefore, we might have missed peer-reviewed papers in other languages and non-peer-reviewed research.

### 4.2. Conclusions

The majority of the current evidence regarding alcohol health warning labels comes from very short interventions in the online or the laboratory setting as opposed to the real world, and there is rather large diversity in the content and format of labels being evaluated. Nevertheless, evidence from real-world, long-term interventions shows that alcohol health warning labels designed to be visible and contain novel and specific information have the potential to be part of an effective labelling strategy. Labelling should be seen as tool to raise awareness and a small part of wider alcohol prevention and policy approach. Future research could focus on importance of labels in a broader societal context, and go beyond individual behaviour change, recognising that change in social norms can be a complex, multifaceted and long-term process.

## Figures and Tables

**Table 1:** Studies overview—aim and methodology.

Authors and Year Published	Country	Study Aim (According to Authors)	Setting	Methodology/Study Design	Sample Characteristics	Population (P) or Inclusion Criteria (IC)	Sampling and Recruitment	Year Data Collected
Al-Hamdani and Smith (2015) [64]	Canada	To apply the lessons learned from the tobacco health warnings and plain packaging literature to an alcohol packaging study and test whether labelling alters consumer perceptions.	Online (survey)	Experiment: 3 × 4 mixed design	N = 92 M(SD)_age_ = 36.4 (13.3) 66.2% female	P: 60.2% students, 39.8% hospital employees 91.1% participants drinking at least weekly	Convenience sample, recruited through posters and ads in the organisations	Not reported
Al-Hamdani and Smith (2017) [76]	Canada	To examine whether increasing the size of HWL and plain packaging lowers ratings of alcohol products and the consumers who use them, increases ratings of bottle “boringness” and enhances warning recognition compared with branded packaging.	Online (survey)	Experiment: 3 × 2 × 3 mixed design	N = 440 initially/241 finally M(SD)_age_ = 26 (7.1) 51.7% female	IC: Adults of legal age who consumed alcohol in the past 12 months P: 91% participants drinking at least weekly	Convenience sample, recruited online	Not reported
Annunziata et al. (2019) [70]	Italy and France	To analyse Generation Y consumers’ preferences for, interest in and attitudes towards different formats of health warnings on wine labels in two countries with different legal approaches: France and Italy.	Online (survey)	Discrete choice experiment	N = 500 (250 per country) M(SD)_age_ = 23.3 (3.4)—FR; 25.2 (4.5)—IT 54% females—FR; 60% females—IT	IC: Generation Y (1978–2000)	Convenience sample, recruited online	2018
Blackwell et al. (2018) [71]	UK	To examine the influence of unit labels and health warnings on drinkers’ understanding, attitudes and behavioural intentions regarding drinking and examine optimal methods of delivering this information on labels.	Online (survey)	Between subjects experimental study	N = 1184 M(SD)_age_ = 35 (12) 50% female	IC: Adult (18+) drinkers only	Recruitment through crowdsourcing platform/panel	Not reported
Clarke, Pechey et al. (2021a) [55]	UK	To obtain a preliminary assessment of the possible impact of (i) image-and-text, (ii) text only and (iii) image-only HWLs on selection of alcoholic versus non-alcoholic drinks.	Online (survey)	Between-subjects experimental study, 2 × 2 factorial design	N = 6024 (completed the study) M(SD)_age_ = 49.5 (15.5)50% female	IC: Adults (18+) who consumed beer or wine regularly (i.e., at least once a week)	Recruited via market research agency	2019
Clarke, Blackwell et al. (2021b) [56]	UK	To estimate the impact of HWLs describing adverse health consequences of excessive alcohol consumption on selection of alcoholic drinks.	Naturalistic shopping laboratory	Between-subjects design	N = 399 M(SD)_age_ = 39.9 (13.7)55% female	IC: Adults (18+) who purchased beer or wine weekly to drink at home	Recruited via market research agency	2020
Glock and Krolak-Schwerdt (2013) [72]	Luxembourg and Germany	Compared the effectiveness of warning labels that contradicted positive outcome expectancies with health-related warning labels.	Laboratory	Between-subjects experimental study, two factorial mixed design	N = 40 M(SD)_age_ = 24.0 (3.2) 60% female	P: Undergraduates, native German speakers 95% drinkers	Recruited through university courses	Not reported
Gold et al. (2020) [63]	UK	To examine whether showing people a health warning alongside our label designs would have a further effect on our secondary outcomes, increasing the perceived risk of alcohol consumption, decreasing the motivation to drink and lowering the level of drinking which people believe to be health-damaging.	Online (survey)	Parallel randomisedcontrolled trial	N = 7516 total/500 for HWL M(SD)_age_ = 44.2 (16.5)50.5% female	IC: English adults (18+) reporting drinking alcohol	Representative sample of the adult population of Englandin terms of age, gender and region, recruited through online panel platform	2019
Hall et al. (2019) [73]	US	To examine US adults’ reactions to health warnings with strong versus weak causal language.	Online (survey)	Between-subjects experimental study	N = 1360 M(SD)_age_ = 37.4 (11.6) 47.3% female	IC: US residents, (18+)	Convenience sample, recruitment via online crowdsourcing platform	2018
Hall et al. (2020) [65]	US	To examine reactions to graphic versus text-only warnings for cigarettes, SSBs and alcohol.	Online (survey)	2 × 2 × 3 Between-subjects experimental study	N= 1352 M_age_ = 37 47% female	IC: US residents, (18+)	Convenience sample, recruitment via online crowdsourcing platform	2018
Hobin, Schoueri-Mychasiw et al. (2020a) [51]	Canada	To examine the effects of strengthening alcohol labels on consumer attention and message processing, and a self-reported reduction in drinking due to the labels.	Real-world	Quasi experimental design	N = 2049 unique cohort participants M(SD)_age_ = intervention 47.4 (14.6); comparison 41.2 (13.7) 50.7% female intervention, 45.1% female control	IC: Adult (19+), current drinkers (at least 1 drink in past 30 days), living in the intervention or comparison cities, bought alcohol at the liquor store and did not self-report being pregnant or breast-feeding	Systematic recruitment -standard intercept technique of approaching every person that passed a pre-identified landmark	2017/2018
Hobin, Weerasinghe et al. (2020b) [52]	Canada	To test the initial and continued effects of cancer warning labels on drinkers’ recall and knowledge that alcohol can cause cancer.	Real-world	Quasi experimental design	N = 2049 unique cohort participants M(SD)_age_ = intervention 47.4 (14.6); comparison 41.2 (13.7) 50.7% female intervention, 45.1% female control	IC: Adult (19+) current drinkers (at least 1 drink in past 30 days), living in the intervention or comparison cities, bought alcohol at the liquor store, did not self-report being pregnant or breast-feeding	Systematic recruitment—standard intercept technique of approaching every person that passed a pre-identified landmark	2017/2018
Hobin, Shokar et al. (2020c) [54]	Canada	To investigate the impact of alcohol labels on (i) unprompted recall of label messages, (ii) depth of cognitive processing of label messages and (iii) self-reported impact on alcohol consumption.	Real-world	Quasi experimental design	N= 1647 unique cohort participants Age: Intervention: 6.8% 19–24, 34.3% 25–44, 58.8% 45 + Control: 12.3% 19–24, 45.5% 25–44, 42.2% 45 + 51.5% female intervention, 44.1% female control	IC: Adult (19+) current drinkers (at least 1 drink in past 30 days), living in the intervention or comparison cities, bought alcohol at the liquor store, did not self-report being pregnant or breast-feeding	Systematic recruitment—standard intercept technique of approaching every person that passed a pre-identified landmark	2017/2018
Jarvis and Pettigrew (2013) [74]	Australia	To assess the effects of warning statements on youth’s alcohol purchase decisions in the context of information relating to proprietary brands and alcohol content.	Online (survey)	Discrete choice experiment	N = 300 Age and gender not reported	IC: 18–25 year old current drinkers of pre-mixed alcoholic beverages, purchased a packaged pre-mixed alcoholic beverage in the past the 4 weeks	Through web panel provider, including equal proportional representation of two age categories	Not reported
Jongenelis, Pettigrew et al. (2018a) [58]	Australia	To examine the effectiveness of messages when delivered by single versus multiple sources.	Online (survey)	Between-subjects experimental study	N = 2087 M(SD)_age_= 36.05 (12.67) 50.1% female	IC: Adult drinkers consuming alcohol at least twice per month	Web panel provider	Not reported
Jongenelis, Pratt et al. (2018b) [59]	Australia	To examine whether exposing at-risk drinkers to warning statements relating to specific chronic diseases increases the extent to which alcohol is believed to be a risk factor for those diseases and influences consumption intentions.	Online (survey)	Between-subjects experimental study	N = 364 18–34 years 29% 35–65 years 71% 28% female	IC: Adults 18–65 years who reported drinking at levels associated with long-term risk of harm (more than 2 SD per day)	Web panel provider	Not reported
Krischler and Glock (2015) [68]	Luxembourg and Germany	To investigate the effectiveness of alcohol warning labels tailored toward young adults’ positive outcome expectancies.	Laboratory	Experiment: 3 × 2 mixed design	N = 122 M(SD)_age_ = 23.5 (3.5) 68.9% female	P: 91.7% Undergraduates	Recruited on campus	2014
Ma (2021) [69]	US	To determine the impact of pictorial warning labels featuring narrative content on risk perceptions and behavioural intentions.	Online (survey)	Between-subjects experimental study	N = 169 M(SD)_age_ = 43.2 (11.5) 37.9% female	IC: Alcohol consumers	Recruited through web panel provider	Not reported
Monk et al. (2017) [66]	UK	To investigate the amount of time spent looking at the different elements of alcohol-related health warnings.	Laboratory	Experiment: 2 × 2 × 2 mixed factorial design	N = 22 M(SD)_age_ = 21.3 (1.7) 68.2% female	University students	Opportunity sampling	Not reported
Morgenstern et al. (2021) [67]	Germany	To investigate impact of alcohol warning labels on knowledge and negative emotions.	Online (survey)	Three factorial experiment	N = 9260 M(SD)_age_ = 12.9 (1.8) 48.6% female	IC: Secondary school students (10–17)	Recruited through schools from randomly selected sub-regions	2017–2018
Pechey et al. (2020) [57]	UK	to describe the potential effectiveness and acceptability of image-and-text (also known as pictorial or graphic) HWLs applied to alcoholic drinks.	Online (survey)	Between-subjects experimental study	N = 5528 M(SD)_age_ = 47.5 (15.8) 50.9% female	IC: 18+, self-reported consuming either beer or wine at least once a week	Purposeful sampling to include range of age, gender and social grades (recruited via market research agency)	2018
Pettigrew et al. (2016) [60]	Australia	To investigate the potential effectiveness of alcohol warning statements designed to increase awareness of the alcohol–cancer link.	Online (survey)	Between-subjects experimental study	N = 1680 < 31 years 49.5 31–45 years 25.2 46–65 years 25.3 49.9% female	IC: Adult (18–65) drinkers, consuming alcohol at least two days per month	Web panel provider	Not reported
Pham et al. (2018) * [50]	Australia	To investigate attention of current in market alcohol warning labels and examine whether attention can be enhanced through theoretically informed design.	Study 1: Online (survey) Study 2: Laboratory	Between-subjects experimental study	Study 1: N = 559 M(SD)_age_ = 31.9 (7.8) Gender not reported Study 2: N = 87 M(SD)_age_ = 26.6 (10.5)Gender not reported	Study 1: P: 72% employed, 6.6% full time students Study 2: P 49.4% full time students, 41.4% employed	Study 1: Snowball recruitment online Study 2: Face to face on campus	2015
Sillero-Rejon et al. (2018) [75]	UK	To examine whether enhancing self-affirmation among a population of drinkers, prior to viewing threatening alcohol pictorial health warning labels, would reduce defensive reactions and promote reactions related to behaviour change, and whether there is an interaction between self-affirmation and severity of warning.	Laboratory	Between-subjects experimental study	N = 128 M(SD)_age_ = 22 (4) Gender not reported	IC: Adult (18+) regular alcohol consumers who have consumed over 14 units per week during the preceding week	Opportunity sampling, recruited via e-mail, posters and websites	Not reported
Stafford and Salmon (2017) [62]	UK	To test whether the speed of alcohol consumption is influenced by the type of alcohol health warning contained on the beverage.	Laboratory	Between-subjects experimental study	N = 45 M(SD)_age_ = 18.9 (1.1) 100% female	IC: Female (18–25) regular alcohol consumers	Recruited using an online system and via social media	Not reported
Wigg and Stafford (2016) [61]	UK	To test the effectiveness of a range of alcohol health warnings, comparing no health warning, text only and pictorial warning.	Laboratory	Between-subjects experimental study	N = 60 M(SD)_age_ = 19.4 (3.1) 71.7% female	IC: Alcohol consumers	Recruited using an online system, received credit points	Not reported
Zhao et al. (2020) [53]	Canada	To test if the labelling intervention was associated with reduced alcohol consumption.	Real-world	Quasi experimental design	Monthly retail sales data, no individual participants	Population 15+ in the research areas	/	2017/2018

* two studies considered as one, as they are studying the same outcome.

**Table 2:** Studied outcomes.

	N	%	Papers
Attitude	11	41%	Al-Hamdani & Smith (2015) [64]; Al-Hamdani & Smith (2017) [76]; Annunziata et al. (2019) [70]; Blackwell et al. (2018) [71]; Glock & Krolak-Schwerdt (2013) [72]; Hall et al. (2020) [65]; Jarvis & Pettigrew (2013) [74]; Jongenelis et al. (2018a) [58]; Krischler & Glock (2015) [68]; Pettigrew et al. (2016) [60]; Stafford & Salmon (2017) [62]
Intentions	8	30%	Clarke et al. (2021a) [55]; Glock & Krolak-Schwerdt (2013) [72]; Jongenelis et al. (2018a) [58]; Jongenelis et al. (2018b) [59]; Krischler & Glock (2015) [68]; Ma (2021) [69]; Pettigrew et al. (2016) [60]; Wigg & Stafford (2016) [61]
Emotion	7	26%	Clarke et al. (2021a) [55]; Clarke et al. (2021b) [56]; Hall et al. (2020) [65]; Ma (2021) [69]; Morgenstern et al. (2021) [67]; Pechey et al. (2020) [57]; Wigg & Stafford (2016) [61]
Acceptability/support	7	26%	Blackwell et al. (2018) [71]; Clarke et al. (2021a) [55]; Clarke et al. (2021b) [56]; Hall et al. (2020) [65]; Hall et al. (2019) [73] Hobin et al. (2020b) [52]; Pechey et al. (2020) [57]
Behaviour	6	22%	Clarke et al. (2021b) [56]; Hobin et al. (2020a) [51]; Hobin et al. (2020c) [54]; Morgenstern et al. (2021) [67]; Stafford & Salmon (2017) [62]; Zhao et al. (2020) [53]
Awareness/recognition	5	19%	Al-Hamdani & Smith (2015) [64]; Al-Hamdani & Smith (2017) [76]; Hobin et al. (2020a) [51]; Hobin et al. (2020b) [52]; Hobin et al. (2020c) [54]
Risk perception	5	19%	Clarke et al. (2021a) [55]; Gold et al. (2020) [63]; Ma (2021) [69]; Sillero-Rejon et al. (2018) [75]; Wigg & Stafford (2016) [61]
Motivation	4	15%	Blackwell et al. (2018) [71]; Gold et al. (2020) [63]; Pechey et al. (2020) [57]; Sillero-Rejon et al. (2018) [75]
Reactance	4	15%	Blackwell et al. (2018) [71]; Clarke et al. (2021a) [55]; Hall et al. (2020) [65]; Sillero-Rejon et al. (2018) [75]
Knowledge	3	11%	Hobin et al. (2020b) [52]; Jongenelis et al. (2018b) [59]; Morgenstern et al. (2021) [67]
Perceived effectiveness	3	11%	Hall et al. (2020) [65]; Hall et al. (2019) [73]; Sillero-Rejon et al. (2018) [75]
Attention	3	11%	Monk et al. (2017) [66]; Pham et al. (2018) [50]; Sillero-Rejon et al. (2018) [75]
Cognitive processing	3	11%	Hall et al. (2020) [65]; Hobin et al. (2020a) [51]; Hobin et al. (2020c) [54]
Avoidance	3	11%	Blackwell et al. (2018) [71]; Clarke et al. (2021a) [55]; Sillero-Rejon et al. (2018) [75]
Efficacy	2	7%	Blackwell et al. (2018) [71]; Hall et al. (2020) [65]

**Table 3:** Results of the studies.

Authors	Studied Groups/Variables (Independent Variables)	Outcome (Dependent Variable)	Category	Significant Results * (< Lower Than, > Higher Than)	Mechanism of Impact (if Tested)
Al-Hamdani & Smith (2015) [64]	Between subjects: standard text warning combined text and image warning combined text and image warning on plain packaged bottles Within subjects: alcohol type: beer, wine and spirits	Product-based perception	Attitude	Plain packaging < Standard (all three alcohol types) Text and image < Standard (all three alcohol types) Text < Image (spirits only)	
		Consumer-based perception	Attitude	Plain packaging < Standard (all three alcohol types) Text and image < Standard (wine and spirits only) No difference text and plain packaging	
		Warning recognition	Awareness/recognition	Higher odds of recognition of warning on plain packaged bottle of wine (but not beer and spirits)	
Al-Hamdani & Smith (2017) [76]	Between subjects: branding type: branded or plain packaged warning size: medium, large, or extra-large Within subjects: alcohol type: beer, wine and spirits	Product-based perception	Attitude	Plain packaging < Branded, no difference in size Interaction between alcohol type and branding and warning size (Extra large < Medium (wine and spirits only)	
		Consumer-based perception	Attitude	Plain packaging < Branded, no difference in size	
		Boringness of the bottle	Attitude	No difference in size or branding Interaction between alcohol type and branding	
		Warning recognition	Awareness/recognition	Higher odds of recognition of warning on plain packaged spirit bottle (but not beer and wine)	
Annunziata et al. (2019) [70]	Attributes: alcohol content framing of warning statement warning size warning position	Utility	Attitude	By attribute importance: No pictorial > Pictorial Back label > Front label No message > Neutral framed Message > Negative framed message	
Blackwell et al. (2018) [71]	Between subjects: message specificity (general vs. specific) message framing (positive vs. negative) message content (cancer vs. mental health)	Support for alcohol labelling	Acceptability/support	Support HWL < Support calorie information and strength information	
		Motivation to drink less	Motivation	Cancer message > Mental health message Negative framing > Positive framing No difference in specificity	
		Reactance	Reactance	Specific message < General message Negative framing > Positive framing No difference in content	
		Avoidance	Avoidance	Cancer message > Mental health message Negative framing > Positive framing No difference in specificity	
		Believability	Attitude	Specific message > General message No difference in framing and content	
		Self-efficacy	Efficacy	No difference in any characteristic	
		Response efficacy	Efficacy	Specific message > General message No difference in framing and content	
Clarke, Pechey et al. (2021a) [55]	Between subjects: image: present versus absent text: present versus absent	Negative emotional arousal	Emotions	Any HWL > No HWL Image and text > Text only	(T) Tested model suggested negative emotional arousal possibly mediates the effect of HWL on alcohol selection
		Acceptability of labels	Acceptability/support	Text > Image and text > Image only	
		Reactance	Reactance	Any HWL > No HWLImage and text > Text only	
		Avoidance	Avoidance	Any HWL > No HWL Image and text > Text only Image > Image and text	
		Perceived disease risk	Risk perception	Any HWL > No HWL No difference between HWLs	
		Proportion of participants selecting an alcoholic beverage to be consumed either immediately or later on that day	Intentions	Any HWL < No label Image or Image and text < Text alone	
Clarke, Blackwell et al. (2021b) [56]	Between subjects: image and text HWL text HWL no HWL (control)	Negative emotional arousal	Emotions	Image and text > text	
		Acceptability of labels	Acceptability/support	Text > Image and text	
		Proportion of total alcoholic drinks among all selected	Behaviour	No difference between the three groups	
Glock & Krolak-Schwerdt (2013) [72]	Between subjects: labels (health-related vs. positive-related) Within subjects: time (before vs. after presentation of warning labels)	Implicit attitudes	Attitude	Interaction between warning and time: Health labels: before < after Positive expectancies: before > after	
		Explicit attitudes	Attitude	No difference	
		Intention to drink	Intentions	Health-related > Positive-related	
Gold et al. (2020) [63]	Between subjects: label: present vs. absent	Perceived personal risk	Risk perception	No difference	
		Motivation to drink less	Motivation	No difference	
		Perception of damaging drinking	Risk perception	No difference	
Hall et al. (2019) [73]	Between subjects: substance: cigarette, alcohol, sugar sweetened beverages causal language: “causes”, “contributes to”, “can contribute to” and “may contribute to”.	Perceived message effectiveness	Perceived effectiveness	Causes > Others (overall, for alcohol less than for cigarettes)	
		Perceived message ineffectiveness	Perceived effectiveness	May contribute to > Others (overall, and for alcohol)	
		Public support	Acceptability/support	No difference in support in alcohol group	
Hall et al. (2020) [65]	Between subjects: label: text vs. image and text efficacy information: present vs. absent Within subjects: substance: cigarette, alcohol, sugar sweetened beverages	Perceived message effectiveness	Perceived effectiveness	Image and text > Text (overall, and for alcohol)	
		Believability	Attitude	Text > Image and text (overall, and for alcohol)	
		Reactance	Reactance	Image and text > Text (overall, and for alcohol)	
		Fear	Emotions	Image and text > Text (overall, and for alcohol)	
		Thinking about harms	Cognitive processing	Image and text > Text (overall, and for alcohol)	
		Product appeal	Attitude	Text > Image and text (overall, and for alcohol)	
		Policy support	Acceptability/support	Text > Image and text (overall, and for alcohol)	
		Self-efficacy	Efficacy	No difference in efficacy information presence	
Hobin, Schoueri-Mychasiw et al. (2020a) [51]	Between subjects: labels: enhanced vs. regular	Recognition of labels	Awareness/recognition	Enhanced label > regular label	
		Cognitive processing	Cognitive processing	Enhanced label > Regular label	(T) Consumer attention to and processing of label messages partially mediated the relationship between the enhanced alcohol warning labels and self-reported drinking less → suggests that strengthening HWL will increase their effectiveness because they draw attention to and increase processing of the labels
		Self-reported impact on drinking (reduction)	Behaviour	Enhanced labels > Regular label	
Hobin, Weerasinghe et al. (2020b) [52]	Between subjects: labels: enhanced vs. regular	Prompted and unprompted recall	Awareness/recognition	Enhanced label > Regular label (largest difference between groups at T2)	
		Knowledge of alcohol as carcinogen	Knowledge	Enhanced label > Regular label (largest difference between groups at T3)	
		Support for health warning labels on alcohol containers	Acceptability/support	Intervention: Agree (Wave 1 = 57.4%; Wave 2 = 57.3%; Wave 3 = 61.3%) Comparison: Agree (Wave 1 = 53.7%; Wave 2 = 51.6%; Wave 3 = 53.7%)	(T) Knowledge about alcohol causing cancer associated with greater likelihood of supporting health warning labels in the study
Hobin, Shokar et al. (2020c) [54]	Between subjects: labels: enhanced vs. regular	Unprompted recall	Awareness/recognition	Enhanced label > Regular label (cancer label)	
		Cognitive processing	Cognitive processing	Enhanced label > Regular label	
		Self-reported impact on drinking (reduction)	Behaviour	Enhanced labels > Regular label	
Jarvis & Pettigrew (2013) [74]	Attributes: brand alcohol content warning message (framed positive or negative)	Utility	Attitude	Brand > Alcohol content > Warning statement Two warning statements significant: one positive and one negative	
Jongenelis, Pettigrew et al. (2018a) [58]	Between subjects: Source: single-source or multiple-source of message	Intentions to reduce alcohol consumption	Intentions	Multiple source > Single source	
		Message believability	Attitude	Multiple source > Single source	
		Message convincingness	Attitude	Multiple source > Single source	
		Personal relevance of the message	Attitude	Multiple source > Single source	
Jongenelis, Pratt et al. (2018b) [59]	Between subjects: Label content	Alcohol as risk factor beliefs	Knowledge	Present message > Absent message (for all messages except for liver damage)	
		Intentions to reduce alcohol consumption	Intentions	Present message > Absent message (for cancer, diabetes and mental illness message, but not for liver damage and heart disease)	
Krischler & Glock (2015) [68]	Between subjects: labels (questions vs. statements vs. control) Within subjects: expectancy category (positive vs. negative, tension reduction vs. negative sedating vs. socially related)	Drinking intentions	Intentions	No difference	
		Individual outcome expectancies	Attitude	No difference	
		General outcome expectancies	Attitude	No difference	
Ma (2021) [69]	Between subjects: narrative PWLs non-narrative PWLs control (no stimulus)	Worry about developing alcohol-related cancer	Emotions	Narrative labels > no labels	
		Feelings of risk of developing alcohol-related cancer	Risk perception	Narrative labels > no labels	
		Comparative likelihood of developing alcohol-related cancer	Risk perception	No difference	
		Perceived severity of harm of developing alcohol-related cancer	Risk perception	Narrative labels > no labels	(T) Mediation analysis found that narrative PWLs (vs. control) indirectly influenced intentions through worry, but not through feelings of risk, comparative likelihood, or perceived severity.
		Intentions to reduce alcohol use	Intentions	No difference	
Monk et al. (2017) [66]	Between subjects: image: graphic or neutral area of interest: text or image positive expectancy change: increase or decrease/no change	Dwell time	Attention	Image > Text (overall and in the positive expectancies increase group)No difference between graphic or neutral, and between increase and decrease in positive expectancies	
Morgenstern et al. (2021) [67]	Between subjects: position of the HWL on the questionnaire (before vs. after alcohol items) type of HWL (text only vs. image and text) content of the HWL (one out of a pool of ten)	Knowledge about alcohol-related risks	Knowledge	Any label >No label (for cancer and liver cirrhosis)	
		Self-report of alcohol use	Behaviour	No difference	
		Negative emotions	Emotions	Text and image > Text only (only for some messages—driving, liver cirrhosis, pharmaceuticals, minors)	
Pechey et al. (2020) [57]	Between subjects: message type (7 messages of different health consequences) image type (3 different graphic images per health consequence)	Negative emotional arousal	Emotions	Bowel cancer > Others (> liver cancer > liver cirrhosis > heart disease > liver disease > 7 types of cancer > breast cancer)	
		Desire to consume the labelled product	Motivation	Bowel cancer < Liver cirrhosis < Breast cancer < Liver cancer < Heart disease < Liver disease < 7 types of cancer	
		Acceptability of the label	Acceptability/support	Low overall acceptability, Bowel cancer < Others (Breast cancer < Liver disease < 7 types of cancer < Liver cancer < Liver disease < Liver cirrhosis)	
Pettigrew et al. (2016) [60]	Between subjects: content of the message (6 messages) Within subjects: before and after (only for intention)	Intention to reduce alcohol consumption	Intentions	Pre < Post No difference between messages	
		Believability of message	Attitude	Bowel cancer > Other messages	
		Convincingness of the message	Attitude	Bowel cancer > Other messages	
		Personal relevance of the message	Attitude	Bowel cancer > Other messages	
Pham et al. (2018) [50]	Between subjects: colour change (use of red colouring instead of black) size of the HWL (increase by 50%) changes in both colour and size control (existing label)	Attention	Attention	Enhanced colour and size > Other groups	
		Number and length of visual fixations	Attention	No difference between groups	
Sillero-Rejon et al. (2018) [75]	Between subjects: self-affirmation (self-affirmed vs. control) Within-subject: warning severity (moderately-severe vs. highly-severe)	Visual attention	Attention	No difference in severity or self-affirmation	
		Avoidance	Avoidance	Highly severe > Moderately severe No difference in self-affirmation	
		Reactance	Reactance	Highly severe > Moderately severe No difference in self-affirmation	
		Perceived susceptibility to health risks	Risk perception	No difference in severity or self-affirmation	
		Perceived warning effectiveness	Perceived effectiveness	Highly severe > Moderately severe No difference in self-affirmation	
		Motivation to drink less	Motivation	Highly severe > Moderate severe No difference in self-affirmation	
Stafford & Salmon (2017) [62]	Between subjects: no HWL text only HWL image and text HWL	Product design evaluation	Attitude	Image and text HWL < No HWL No difference between text only and no HWL	(H) The mechanism responsible for slower consumption is theorised to be due to higher levels of fear arousal in the two health warning conditions
		The duration to consume the test beverage	Behaviour	No HWL < Text HWL No HWL < Image and text HWL No difference between text HWL and image and text HWL	
Wigg & Stafford (2016) [61]	Between subjects: no HWL text only HWL image and text HWL	Fear arousal	Emotions	Image and text HWL > Text HWL Image and text HWL > No HWL No difference between text HWL and no HWL	
		Risk perception	Risk perception	Image and text HWL > No HWL No difference between text HWL and no HWL No difference between image and text HWL and text HWL	
		Intention to quit/reduce alcohol consumption	Intentions	Image and text HWL > No HWL No difference between text HWL and no HWL No difference between image and text HWL and text HWL	
Zhao et al. (2020) [53]	Between subjects: label: enhanced vs. regular	Alcohol sales	Behaviour	Enhanced labels < Regular labels	

* Significant results column describes the impact of independent variables (tested groups) on the dependent variable (outcome) and should be read together with the outcome column, for example, “plain packaging group had significantly lower product based perception compared to standard packaging group”.

**Table 4:** Research and policy implications.

**Future Labelling Research**
○ Aim to be explicit about the theory of change underpinning the research and adjust the studied outcomes accordingly. ○ Develop labels to be tested with own qualitative research and preferably co-created with local stakeholders/populations—focus on both content and format. ○ The information provided in the labels should be clear and reflect the up-to-date literature on alcohol-attributable burden of disease. ○ Online surveys with large samples and experimental design can be a good lower-cost approach to help with tailoring the messages to new contexts before testing them in the real world. ○ Real-world studies with longer-term outcomes are preferable in the context a lot of existing research (e.g., online and lab surveys). ○ When reporting, describe the context in which the intervention was conducted; frameworks such as Medical Research Councils’ process evaluation guidance [82] can be applied to support design and reporting.
**Development of mandatory labels**
As currently implemented mandatory and voluntary labels are often suboptimal, existing research on new health warning labels points to the following characteristics: ○ In terms of format: ○ good visibility of the label is essential: this is achieved by large size, contrasting colours, large text, prominent position on the product ○ In terms of content: ○ it is important to have messages that convey novel information to the target population ○ messages should be evidence-based—for example, take into account alcohol-attributable risk, morbidity and mortality ○ wording should be simple, messages should be specific rather than general ○ current evidence suggests that graphic images are not well accepted, and does not point to a superiority of their inclusion ○ use rotating health warning labels presented in combination with other messages ○ information on health warning labels should be presented also through other sources of information (e.g., information campaigns), and labelling should preferably also be introduced as part of a broader package of alcohol policy measures

**Figure A1:**
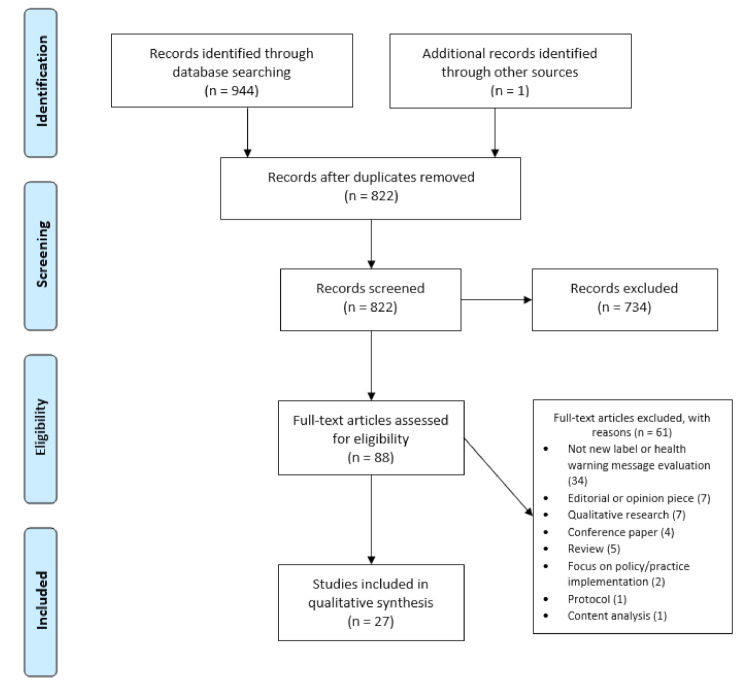
PRISMA Diagram.

## Data Availability

Extracted data are presented in the main and supplementary tables.

## Supplementary Materials

The following are available online at https://www.mdpi.com/article/10.3390/nu13093065/s1, Table S1. Label development, content and format.
