# Supplementary material for: Nature and Potential Impact of Alcohol Health Warning Labels: A Scoping Review
Source: Nutrients. 2021 Aug 31;13(9):3065. doi: 10.3390/nu13093065 (PMC8469468; doi:10.3390/nu13093065)
Supplement: Supplementary file 1 [file nutrients-13-03065-s001.zip › nutrients-1328533-supplementary.pdf]

Table S1: Label development, content and format

	Label content		Label format	How was label developed	How presented	Duration of presentation	Alcohol type	Language of warning
	Text	*Image*						
Al-Hamdani & Smith (2015)	Heavy drinking causes liver cancer. Your chances for a 5-year survival from the disease are 3% when caught in its late stages	Liver cancer (diseased image)	Depending on condition:<br>Text: occupying 25% of the front body area<br>Text and image: 50% of the front body area<br>Plain package: 50% of the front body area, removed brand imagery items and using standardized font	Developed by researchers, based on tobacco health warnings literature	Picture of label on the bottle	Very short	Beer, wine and hard liquor	English
Al-Hamdani & Smith (2017)	Heavy drinking causes liver cancer. Your chances for a 5-year survival from the disease are 3% when caught in its late stages	Liver cancer (diseased image)	Depending on condition: occupying 50, 75% or 90% of front body area<br>Plain package: removed brand imagery items and using standardized font	Developed by researchers, not explicitly mentioned how	Picture of label on the bottle (on the screen)	Very short	Beer, wine and hard liquor	English
Annunziata et al (2019)	Each alcoholic beverage damages your brain (long term effect)<br><br>For your safety, don't drink and drive (short term effect)	Congruent pictorial	Logo position: back or front<br>Logo size: big or small	Developed by researchers, based on others' previous research	Image of bottle with label (on the screen)	Very short	Wine	Italian, French
Blackwell et al (2018)	Alcohol increases your risk of cancer/mental illness (general, negative)<br>Drinking less reduces your risk of cancer/mental illness (general, positive)<br>Alcohol increases your risk of bowel cancer/depression (specific, negative)<br>Drinking less reduces your risk of bowel cancer/depression (specific, positive)	/	Black text on white background	Developed by researchers, Based on own and others' previous alcohol HWL research	Image of label on the bottle (on the screen)	Very short	Beer	English
Clarke et al (2021a)	Alcohol causes bowel cancer<br>Alcohol causes breast cancer<br>Alcohol causes liver cancer	Realistic photo of diseased organ	Black text on white background with black border, rotating messages	Developed by researchers, based on alcohol harm research, labelling research, and own previous labelling research	Picture of label on the bottle (on the screen)	Very short	Depending on previously established preference (beer or wine)	English

Clarke et al (2021b)	Alcohol causes bowel cancer<br>Alcohol causes breast cancer<br>Alcohol causes liver cancer<br>Alcohol causes liver cirrhosis<br>Alcohol causes heart disease	Realistic photo of diseased organ	Black text on white background with black border, rotating messages	Developed by researchers, based on previous alcohol HWL research	Labels on products in the lab store	Very short	All drinks for the shopping,<br><br>Post-shopping survey: beer or wine, depending on previously established preference	English
Glock & Krolak-Schwerdt (2013)	Health-related:<br>Alcohol damages your brain<br>Alcohol endangers your health,<br>Alcohol damages your cardiovascular system,<br>Alcohol damages your liver,<br>Alcohol drinking is highly addictive<br><br>Positive expectancies:<br>Alcohol reduces your capacity,<br>Alcohol does not reduce your tension,<br>Alcohol leads to problems with other people,<br>Alcohol increases stress,<br>Alcohol makes you feel alone	/	Front of pack, large font	Developed by researchers, on previous positive expectancy research	Picture of label on the bottle (on the screen)	Very short	Wine	German
Gold et al (2020)	Warning: Alcohol causes cancer	/	In bold type – black text on white background, with red border	Not mentioned	Picture of the label next to the bottle (on the screen)	Very short	Beer, wine and spirits	English
Hall et al (2019)	WARNING: Drinking alcohol [causal language variant] liver disease	/	Not mentioned	Developed by researchers, Based on alcohol harm research	Picture of the label (on the screen)	Very short	Beer	
Hall et al (2020)	WARNING: Drinking alcohol causes liver disease<br><br>Efficacy information - Cut back: www.cutback.gov	Copyright-free images depicting diseased body parts	White text on black background, size depending on condition	Developed by researchers, based on alcohol harm research	Picture of the label on the bottle – mock brand (on the screen)	Very short	Beer	English
Hobin et al (2020a)	Alcohol can cause cancer, including breast and colon cancers<br><br>(additionally: national drinking guidelines and standard drinks contained in the bottle)	/	Large, bright yellow background and red border, rotating messages, pre-determined size	Developed by researchers and in consultation with stakeholders, based on own and others' previous research	Label stickers on the bottle in the store	One month cancer label, additional 3,5 months other labels	All except select local and single serve beer and cider	English and French

Hobin et al (2020c)	Alcohol can cause cancer, including breast and colon cancers<br><br>(additionally: drinking guidelines and standard drinks contained in the bottle)	/	Large, bright yellow background and red border, rotating messages, pre-determined size	Developed by researchers and in consultation with stakeholders, based on own and others' previous research	Label stickers on the bottle in the store	One month cancer label, additional 3,5 months other labels	All except select local and single serve beer and cider	English and French
Hobin et al (2020b)	Alcohol can cause cancer, including breast and colon cancers<br><br>(additionally: drinking guidelines and standard drinks contained in the bottle)	/	Large, bright yellow background and red border, rotating messages, pre-determined size	Developed by researchers and in consultation with stakeholders, based on own and others' previous research	Label stickers on the bottle in the store	One month cancer label, additional 3,5 months other labels	All except select local and single serve beer and cider	English and French
Jarvis & Pettigrew (2013)	Keep your brain healthy (Positive)<br>Every drink of alcohol harms your brain (Negative)<br>Make sure you are okay to drive (Positive)<br>Drunk driving kills (Negative)	/	No specific formatting	Developed by researchers based on own qualitative research	Message next to brand name	Very short	Pre-mixed drinks	English
Jongenelis et al (2018a)	Alcohol increases your risk of bowel cancer<br>Alcohol increases your risk of breast, bowel, throat, and mouth cancer<br>Alcohol increases your risk of breast cancer<br>Warning: Alcohol increases your risk of cancer<br>Alcohol increases your risk of cancer<br>Reduce your drinking to reduce your risk of cancer	/	No specific formatting<br><br>Exposed to the statements via an advertisement in a newspaper, a warning on an alcohol product, a comment made by a child about information learned during a health class at school, on a billboard, in a message given by a doctor	Developed by researchers based on own multistage research	In a computer simulation (label on the bottle)	Very short	Depending on previously established preference (beer, wine, spirits/liquor)	English
Jongenelis et al (2018b)	Warning: Alcohol increases your risk of cancer<br>Warning: Alcohol increases your risk of diabetes<br>Warning: Alcohol increases your risk of liver damage<br>Warning: Alcohol increases your risk of mental illness<br>Warning: Alcohol increases your risk of heart disease	/	No specific formatting	Developed by researchers based on own multistage research	In a computer simulation (label on the bottle)	Very short	Depending on previously established preference (beer, wine, spirits/liquor)	English
Krischler & Glock (2015)	Question:<br>Do you really want alcohol to help you loosen your inhibitions?<br>Do you really want alcohol to help you meet new people?	Photos associated with text	Black text on white background	Developed by researchers based on own research	label on the bottle (in the lab)	Very short	Beer and alcopops	German

	Do you really want alcohol to help you test your limits?<br><br>Statement:<br>Yes, alcohol helps loosen your inhibitions<br>Yes, alcohol helps you meet new people<br>Yes, alcohol helps you test your limits							
Ma (2021)	Narrative:<br>Alcohol gave me bowel cancer<br>Alcohol gave me liver cancer<br><br>Non-narrative:<br>Alcohol causes bowel cancer<br>Alcohol causes liver cancer	Narrative PWL: cancer patient photo<br><br>Non-narrative: diseased body part image	Black text on white background, large part of bottle	Developed by researchers based on others' research	Image of label on the bottle (on the screen)	Very short	Beer and wine	English
Monk et al (2017)	50 warnings in total<br>(e.g, alcohol affects brain functioning and alcohol causes liver disease)	Content congruent image; graphic (explicit medical images) or neutral (cartoon physiology)	White square	Developed by researchers based on others' labelling research	Image of label only (on the screen)	Very short	None (label only)	English
Morgenstern et al (2021)	Alcohol may harm the unborn baby<br>Alcohol can cause liver cirrhosis<br>Alcohol can cause mental health problems<br>Alcohol can cause dependence<br>Alcohol can cause cancer<br>Don't drink when taking medicine<br>Alcohol may harm the unborn baby<br>Alcohol slows your reaction time – don't drink and drive<br>Don't serve alcohol to minors<br>Don't drink while operating machinery	Congruent images	Red text on white background with red border	Labels developed by NGO	Image of label only (on the screen, on the paper)	Very short	None (label only)	German
Pechey et al (2020)	Alcohol causes liver cancer<br>Alcohol causes bowel cancer<br>Alcohol causes liver cirrhosis<br>Alcohol causes breast cancer<br>Alcohol causes liver disease<br>Alcohol causes 7 types of cancers<br>Alcohol causes heart disease	Graphic image depicting the health outcome described in the text	Black text on white background with black border	Developed by researchers based on previous research, evidence about designing effective messages, own piloting of images	Image of label on the screen	Very short	Beer or wine, depending on stated preference	English
Pettigrew et al (2016)	Alcohol increases your risk of bowel cancer<br>Warning: Alcohol increases your risk of cancer<br>Alcohol increases your risk of cancer	/	Not mentioned	Developed by researchers based on own research	In a computer simulation (label on the bottle)	Very short	Depending on previously established preference	English

	Alcohol increases your risk of breast, bowel, throat and mouth cancer<br>Alcohol increases your risk of breast cancer<br>Reduce your drinking to reduce your risk of cancer							
Pham et al (2018)	Drinkwise slogan: Get the facts: Drinkwise.org.au	Pregnancy pictogram	Depending on condition: colour (use of red colouring instead of black); changing the size of the warning labels (increase of warning label size by 50%); changes in both colour and size of the warning labels	Enhancing existing voluntary labels based on theory	Study 1: Message on the screen<br><br>Study 2: Image of label only (on the screen)	Very short	1st: None<br><br>2nd: wine	English
Sillero-Rejon et al (2018)	Alcohol causes liver cirrhosis<br>Alcohol damages your brain<br>Alcohol is associated with depression and anxiety<br>Alcohol damages your unborn baby<br>Drinking alcohol causes road accidents<br>Alcohol causes cancer	Moderate or graphic (e.g. diseased organ) congruent image	White and red text on black background, bottom third of the bottle	Developed by researchers, based on recommendations from other researchers and NGO	Image of branded can with label (on the screen)	Very short	Beer	English
Stafford & Salmon (2017)	Alcohol causes fatal liver cancer	Diseased liver	Front of pack, black text on white background with black border	Based on design used in another study	Label on the actual bottle in the lab		Alcopop	English
Wigg & Stafford (2016)	Alcohol causes fatal liver cancer	Diseased liver	Front of pack, black text on white background with black border	Developed by researchers, based on own preliminary study	Label on the actual bottle, foreign brand	Very short	Beer and wine	English
Zhao et al (2020)	Alcohol can cause cancer, including breast and colon cancers<br><br>(additionally: drinking guidelines and standard drinks contained in the bottle)	/	Large, bright yellow background and red border, rotating messages, pre-determined size	Developed by researchers and in consultation with stakeholders, based on own and others' previous research	Label stickers on the bottle in the store	One month cancer label, additional 3,5 months other labels	All except select local and single serve beer and cider	English and French
